# Genome-wide association study of hemolytic uremic syndrome causing Shiga toxin-producing *Escherichia coli* from Sweden, 1994–2018

**DOI:** 10.1007/s10096-023-04600-1

**Published:** 2023-04-27

**Authors:** Andreas Matussek, Sara Mernelius, Milan Chromek, Ji Zhang, Anne Frykman, Sverker Hansson, Valya Georgieva, Yanwen Xiong, Xiangning Bai

**Affiliations:** 1 Department of Microbiology, Division of Laboratory Medicine, Oslo University Hospital, Oslo, Norway; 2 Department of Microbiology, Division of Laboratory Medicine, Institute of Clinical Medicine, University of Oslo, Oslo, Norway; 3 Division of Clinical Microbiology, Department of Laboratory Medicine, Karolinska Institutet, Stockholm, Sweden; 4 Laboratory Medicine, Department of Clinical and Experimental Medicine, Jönköping Region County, Linköping University, Jönköping, Sweden; 5 Department of Laboratory Medicine, Jönköping, Sweden; 6 Division of Pediatrics, Department of Clinical Science, Intervention and Technology, Karolinska Institutet and Karolinska University Hospital, Stockholm, Sweden; 7 Fonterra Research and Development Centre, Dairy Farm Road, Palmerston North, New Zealand; 8 Department of Pediatrics, Institute of Clinical Sciences, Sahlgrenska Academy, University of Gothenburg, Gothenburg, Sweden; 9 Queen Silvia Children’s Hospital, Sahlgrenska University Hospital, Gothenburg, Sweden; 10 State Key Laboratory of Infectious Disease Prevention and Control, Chinese Center for Disease Control and Prevention, National Institute for Communicable Disease Control and Prevention, Beijing, China

**Keywords:** Shiga toxin-producing *Escherichia coli*, Hemolytic uremic syndrome, Pathogenicity, Whole genome sequencing, Genome-wide association study

## Abstract

Shiga toxin-producing *Escherichia coli* (STEC) infection can cause clinical manifestations ranging from diarrhea to potentially fatal hemolytic uremic syndrome (HUS). This study is aimed at identifying STEC genetic factors associated with the development of HUS in Sweden. A total of 238 STEC genomes from STEC-infected patients with and without HUS between 1994 and 2018 in Sweden were included in this study. Serotypes, Shiga toxin gene (*stx*) subtypes, and virulence genes were characterized in correlation to clinical symptoms (HUS and non-HUS), and pan-genome wide association study was performed. Sixty-five strains belonged to O157:H7, and 173 belonged to non-O157 serotypes. Our study revealed that strains of O157:H7 serotype especially clade 8 were most commonly found in patients with HUS in Sweden. *stx2a* and *stx2a* + *stx2c* subtypes were significantly associated with HUS. Other virulence factors associated with HUS mainly included intimin (*eae*) and its receptor (*tir*), adhesion factors, toxins, and secretion system proteins. Pangenome wide-association study identified numbers of accessory genes significantly overrepresented in HUS-STEC strains, including genes encoding outer membrane proteins, transcriptional regulators, phage-related proteins, and numerous genes related to hypothetical proteins. Whole-genome phylogeny and multiple correspondence analysis of pangenomes could not differentiate HUS-STEC from non-HUS-STEC strains. In O157:H7 cluster, strains from HUS patients clustered closely; however, no significant difference in virulence genes was found in O157 strains from patients with and without HUS. These results suggest that STEC strains from different phylogenetic backgrounds may independently acquire genes determining their pathogenicity and confirm that other non-bacterial factors and/or bacteria-host interaction may affect STEC pathogenesis.

**Supplementary Information:**

The online version contains supplementary material available at 10.1007/s10096-023-04600-1.

## Introduction

Shiga toxin-producing *Escherichia coli* (STEC) represents a diverse group of *E. coli* producing one or two different types of Shiga toxin (Stx) [1]. STEC infection causes clinical manifestations ranging from mild, watery diarrhea to bloody diarrhea with severe abdominal pain (hemorrhagic colitis), and potentially fatal hemolytic uremic syndrome (HUS) characterized by the triad of non-immune hemolytic anemia, thrombocytopenia, and acute kidney injury. It has been reported that 5–15% of STEC cases progress to HUS [2, 3]. O157:H7 has been considered as the most common serotype associated with severe disease such as HUS. In recent years, the emerging clinical importance of non-O157 serotypes has been noted primarily due to the improvements in diagnostic tests [4–6].

The key STEC virulence factor Stx encoded by *stx* located on bacteriophages can damage intestinal, vascular, and renal cells leading to gastrointestinal and renal diseases [7]. There are two immunologically distinct Stx types, i.e., Stx1 and Stx2, which can be further divided into various subtypes [8]. Different Stx subtypes display dramatic differences in potency [9]. The presence of *stx2* especially *stx2a* subtype (with and without *stx2c*) correlates highly with the development of HUS, whereas, other Stx1/Stx2 subtypes are linked to mild symptoms [10]. Stx production is essential but not sufficient for STEC virulence. The majority of pathogenic STEC strains, particularly O157:H7 serotype, possess a pathogenicity island known as the locus of enterocyte effacement (LEE), which encodes genes involved in effacement of intestinal epithelial cell microvilli and in intimate adherence between bacteria and the epithelial cell membrane [11]. The major virulence factors encoded on the LEE are intimin (encoded by *eae*), translocated intimin receptor (*tir*), and a type III secretion system [11]. STEC strains harbor additional virulence genes that influence their pathogenic potential, such as *astA* (enteroaggregative *E. coli* heat-stable toxin 1), *toxB* (cytotoxin), *ehxA* (enterohemolysin), and non-LEE encoded adherence genes [12]. The molecular mechanism underlying the pathogenicity among diverse STEC strains remains to be further elucidated.

Previous epidemiological studies have evaluated the risk of development of STEC-associated HUS in correlation to serotypes, *stx* subtypes, and other virulence factors in STEC strains from Nordic countries such as Finland, Norway, and Denmark [13–16], with various results. In Sweden, we have previously analyzed a collection of STEC strains from patients with HUS in correlation to clinical outcomes in HUS patients [17] and also strains from STEC-infected patients with/without bloody diarrhea [18], yet, a comparative study between HUS-STEC and non-HUS-STEC strains is lacking in Sweden. Herein, we performed a genome-wide association study on all clinical STEC strains isolated from patients with and without HUS in Sweden between 1994 and 2018, with the aim to identify genetic factors of STEC predicting the potential to cause HUS.

## Materials and methods

### Collection of STEC isolates and whole genome data

STEC isolates were collected from STEC-infected patients with and without HUS in three regions in Sweden between 1994 and 2018; clinical characteristics were described previously [17, 18]. Metadata of all STEC isolates used in this study are present in Supplementary Table S1. Genome assemblies of STEC isolates were accessible with accession numbers presented in Supplementary Table S1.

### Molecular characterization of STEC isolates

Characterization of *stx* subtypes, serotypes, and virulence genes of all isolates were performed as previously described [17, 18]. In brief, an in-house *stx* subtyping database including all identified *stx1/stx2* subtypes was created as recently described [16]; genome assemblies were compared against the *stx* subtyping database, SerotypeFinder database (https://cge.food.dtu.dk/services/SerotypeFinder), and VFDB database (http://www.mgc.ac.cn/VFs/) to determine the *stx* subtypes, serotypes, and virulence factors genes, respectively, using ABRicate version 1.0.1 (https://github.com/tseemann/abricate) with default parameters. The clade 8-specific SNP in O157:H7 strains was detected by scanning the genome assemblies using an *in-house* program (https://github.com/jizhang-nz/clade8) [19].

Fisher’s exact test using R software version 4.1.1 (https://www.r-project.org) was used to assess association between *stx* subtypes/serotypes/virulence genes and HUS status; Benjamini–Hochberg method was used to adjust p values in the case of multiple testing. *stx* subtypes/serotypes/virulence genes with Benjamini–Hochberg adjusted *p* value below 0.05 were considered statistically significantly associated with HUS or non-HUS.

### Pangenome-wide association study (PWAS)

Genome assemblies were annotated using Prokka v1.14.6 [20]; pangenomes of all STEC isolates were then calculated from genome annotations using Roary (https://github.com/sanger-pathogens/Roary) [21] with the command: roary -s -e -mafft *.gff. Pangenomes consist of a complete set of core and accessory genes in all analyzed isolates [22]. In this study, core genes are defined as genes present in ≥ 99% of isolates; the remaining were classified as accessory (noncore) genes. Associations between the presence/absence of accessory genes and HUS *vs.* non-HUS symptoms were analyzed using Scoary v1.6.16 (run with 1,000 permutation replicates) [23]. Accessory genes were reported as statistically significantly associated with HUS or non-HUS if they attained a Benjamini–Hochberg adjusted *p* value below 0.05. Multiple correspondence analysis (MCA) of pangenomes was performed using the “gene_presence_absence” table generated from Roary as previously described [18]. The R function MCA from R package FactoMineR was used for the analysis [24].

### Whole-genome phylogenetic analysis

Whole-genome multilocus sequence typing (wgMLST) and whole-genome phylogeny analysis were performed to assess phylogenetic relatedness of STEC isolates from patients with and without HUS. To define wgMLST allelic profiles, Fast-GeP (https://github.com/jizhang-nz/fast-GeP) [25] with default settings was performed. The complete genome sequence of O157:H7 strain Sakai (NC_002695.2) was used as a reference. The whole-genome polymorphic sites–based phylogeny was inferred from the concatenated sequences of the coding sequences shared by all genomes. All the regions with elevated densities of base substitutions were eliminated, and a final Maximum Likelihood tree was generated by Gubbins (version 2.3.4) [26] with default settings. The phylogenetic tree was annotated using on online tool ChiPlot (https://www.chiplot.online/).

## Results

### Molecular characteristics of STEC isolates in correlation to HUS

A total of 238 STEC isolates from patients with HUS (*n* = 59) and without HUS (*n* = 179) were included in this study. Out of 238 isolates, 184 were isolated from STEC-infected individuals from 2003 through 2017 in Region Jönköping County, Sweden; five of them developed HUS. Fifty-four were isolated from patients with HUS in Gothenburg and Stockholm, Sweden, from 1994 through 2018. Fifty-four serotypes were identified among all STEC isolates, with O157:H7 being the most predominant serotype (27.3%, 65/238), followed by O26:H11 (16%, 38/238), O121:H19 (10.9%, 26/238), and O103:H2 (8%, 19/238) (Table 1). O157:H7 was significantly overrepresented in HUS-STEC strains (54.2%, 32/59) compared to non-HUS-STEC strains (18.4%, 33/179) (adjusted *p* < 0.001). Out of 65 O157:H7 strains, 46 belonged to clade 8. All HUS-associated O157:H7 strains (*n* = 32), except one, belonged to clade 8 (Supplementary Table S1).

**Table 1:** Serotypes and *stx* subtypes of STEC in correlation to HUS and non-HUS status

	HUS (*n* = 59)	Non-HUS (*n* = 179)	Adjusted *p*
	No. of isolate (%)	No. of isolates (%)	
Serotype			
O157:H7	32 (54.2)	33 (18.4)	< 0.001^*^
O26:H11	4 (6.8)	34 (19.0)	0.142
O121:H19	11 (18.6)	15 (8.4)	0.173
O103:H2	1 (1.7)	18 (10.1)	0.173
O111:H8	2 (3.4)	3 (1.7)	1
O117:H7	0	5 (2.8)	0.715
O104:H4	2 (3.4)	2 (1.1)	0.625
O150:H10	0	4 (2.2)	1
O165:H25	2 (3.4)	2 (1.1)	0.625
O113:H4	0	3 (1.7)	1
O123:H2	0	3 (1.7)	1
O128ab:H2	0	3 (1.7)	1
O145:H28	1 (1.7)	2 (1.1)	1
O146:H21	0	3 (1.7)	1
O177:H25	0	3 (1.7)	1
O91:H21	0	3 (1.7)	1
Others	4 (6.8)	43 (24.0)	0.035*
*stx* subtype			
*stx1a*	3 (5.1)	70 (39.1)	< 0.001^*^
*stx2a* + *stx2c*	29 (45.2)	19 (10.6)	< 0.001^*^
*stx2a*	22 (37.3)	24 (13.4)	0.001^*^
*stx2c*	3 (5.1)	11 (6.1)	1
*stx1a* + *stx2a*	2 (3.4)	7 (3.9)	1
*stx1c*	0	9 (5.0)	0.498
*stx2b*	0	8 (4.5)	0.499
*stx1c* + *stx2b*	0	8 (4.5)	0.499
*stx1a* + *stx2c*	0	8 (4.5)	0.499
*stx2d*	0	4 (2.2)	1
*stx2e*	0	2 (1.1)	1
*stx2g*	0	2 (1.1)	1
*stx1a* + *stx2d*	0	2 (1.1)	1
*stx1a* + *stx2b*	0	2 (1.1)	1
*stx2b* + *stx2d*	0	1 (0.6)	1
*stx1c* + *stx2d*	0	1 (0.6)	1
*stx1d*	0	1 (0.6)	1

^*^Statistically significant difference (Benjamini–Hochberg adjusted *p* < 0.05)

Seventeen *stx* subtypes/combinations were detected, the predominant *stx* subtypes were *stx1a* (30.7%, 73/238), *stx2a* + *stx2c* (20.2%, 48/238), *stx2a* (19.3%, 46/238), *stx2c* (5.9%, 14/238), *stx1a* + *stx2a*, and *stx1c* (3.8%, 9/238), among which, *stx2a* + *stx2c* and *stx2a* were significantly more prevalent in HUS-STEC strains, while *stx1a* was significantly overrepresented in non-HUS-STEC strains (Benjamini–Hochberg adjusted *p* < 0.05) (Table 1).

In addition to *stx*, numerous additional virulence genes were identified to be significantly different between HUS-STEC group and non-HUS-STEC group (Benjamini–Hochberg adjusted *p* < 0.05) (Supplementary Table S2). Virulence genes significantly overrepresented in HUS-STEC strains included genes encoding intimin (*eae*) and its receptor (*tir*); adhesion factor (*paa*); toxins such as cytotoxin (*toxB*) and enteroaggregative heat-stable enterotoxin 1 (*astA*); type III secretion system proteins; and others (Table 2). We performed further statistical analysis on virulence genes in 65 O157:H7 strains; no virulence gene was significantly overrepresented in O157:H7 strains from HUS patients compared to strains from non-HUS patients.

**Table 2:** Virulence genes significantly overrepresented in STEC strains from HUS patients compared to strains from non-HUS patients^#^

Function	Gene^*^	HUS (*n* = 59)	Non-HUS (*n* = 179)	Function	Gene^*^	HUS (*n* = 59)	Non-HUS (*n* = 179)
		No. of isolates (%)	No. of isolates (%)			No. of isolates (%)	No. of isolates (%)
Adherence	*eae*	56 (94.9)	118 (65.9)	Type III secretion system	*escG/S*	56 (94.9)	119 (66.5)
	*tir*	33 (55.9)	35 (19.6)		*sepD*	56 (94.9)	119 (66.5)
	*paa*	55 (93.2)	114 (63.7)		*nleF*	54 (91.5)	110 (61.5)
Toxin	*toxB*	48 (81.4)	81 (45.3)		*nleL*	52 (88.1)	112 (62.6)
	*astA*	34 (57.6)	44 (24.6)		*espN*	52 (88.1)	111 (62.0)
Autotransporter	*espP*	51 (86.4)	90 (50.3)		*espW*	52 (88.1)	106 (59.2)
Type III secretion system	*espJ*	45 (76.3)	55 (30.7)		*espH*	52 (88.1)	114 (63.7)
	*nleB*	39 (66.1)	43 (24.0)		*nleD*	34 (57.6)	67 (37.4)
	*espL*	47 (79.7)	69 ( 38.5)		*espK*	48 (81.4)	112 (62.6)
	*espO1*	53 (89.8)	91 (50.8)	Type VI secretion system	*fha*	53 (89.8)	115 (64.2)
	*espB*	37 (62.7)	40 (22.3)		*tssG*	53 (89.8)	116 (64.8)
	*espR*	33 (55.9)	33 (18.4)		*tssF*	53 (89.8)	118 (65.9)
	*espM*	52 (88.1)	91 (50.8)		*tssC*	53 (89.8)	123 (68.7)
	*nleA*	52 (88.1)	92 (51.4)		*tssB*	53 (89.8)	124 (69.3)
	*espX/Z*	33 (55.9)	35 (19.6)		*hcp2*	53 (89.8)	124 (69.3)
	*espY*	33 (55.9)	36 (20.1)		*clpV*	52 (88.1)	124 (69.3)
	*nleH*	55 (93.2)	108 (60.3)	Others	*shuS*	33 (55.9)	41 (22.9)
	*escC*	56 (94.9)	116 (64.8)		*shuA*	32 (54.2)	41 (22.9)
	*escD*	56 (94.9)	117 (65.4)		*chuU*	33 (55.9)	47 (26.3)
	*espA/D*	56 (94.9)	118 (65.9)		*shuY*	33 (55.9)	35 (19.6)
	*espG*	56 (94.9)	117 (65.4)		*shuT*	33 (55.9)	36 (20.1)
	*etgA*	56 (94.9)	118 (65.9)		*shuX*	33 (55.9)	38 (21.2)
	*map*	56 (94.9)	118 (65.9)		*cesAB/D/L/T*	56 (94.9)	118 (65.9)
	*nleC*	50 (84.7)	90 (50.3)		*escN*	56 (94.9)	117 (65.4)
	*nleE*	56 (94.9)	115 (64.2)		*chuV/W*	33 (55.9)	47 (26.3)
	*sepL*	56 (94.9)	118 (65.9)		*cesF*	53 (89.8)	117 (65.4)
	*escE*	56 (94.9)	118 (65.9)		*aslA*	33 (55.9)	51 (28.5)
	*escQ*	56 (94.9)	117 (65.4)		*stcE*	32 (54.2)	55 (30.7)

^#^All virulence genes significantly different (Benjamini–Hochberg adjusted *p* < 0.05) between HUS-STEC and non-HUS-STEC strains are shown in Supplementary Table S2. ^*^The protein encoded by each gene is presented in Supplementary Table S2

### PWAS of STEC strains from patients with and without HUS

**Fig. 1:**
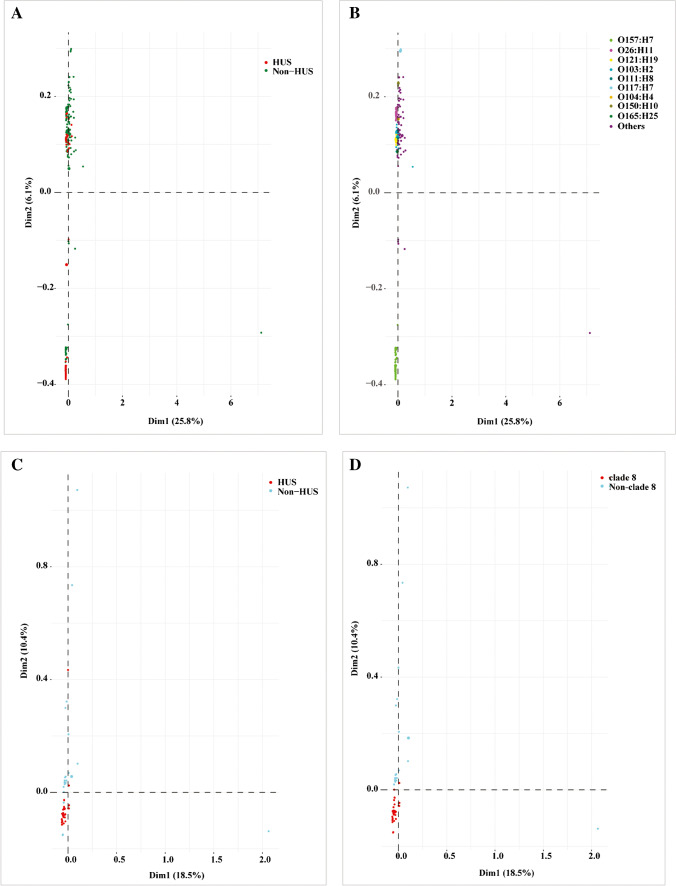
Multiple correspondence analysis plot comparing pangenomes of Shiga toxin-producing *Escherichia coli* (STEC) isolates in this study. All strains (*n* = 238) from patients with HUS and non-HUS are indicated by the red and green rings, respectively (A), the main serotypes are shown in different colors (B). O157:H7 strains (*n* = 65) from patients with HUS and non-HUS are indicated by the red and blue rings, respectively (C), the clade 8 status of O157:H7 strains is displayed (D)

A total of 19,059 genes were identified in the pangenomes of 238 STEC strains using Roary. Scoary identified 954 accessory genes that were significantly overrepresented in HUS-STEC group compared to non-HUS-STEC group (Benjamini–Hochberg adjusted *p* < 0.05) (Supplementary Table S3). The majority of these significant genes, including 12 unique genes in HUS-STEC group, encoded hypothetical proteins (HP) based on annotation using Prokka. The functionally-characterized significant genes overrepresented in HUS-STEC group encoded intimin (*eae*) and its receptor (*tir*), adhesin proteins (*yfcP*, *yehD*, *elfG*, *sfmA*, etc.), and secretion system factors, in line with virulence genes characterization. In addition, genes encoding outer membrane proteins, transcriptional regulators, phage-related proteins, etc., were significantly more prevalent in HUS-STEC group (Supplementary Table S3). MCA of pangenomes separated O157:H7 strains from non-O157 strains, while no distinct cluster was observed for HUS-STEC group (Fig. 1A and 1B).

PWAS was further performed on 65 O157:H7 strains to identify any accessory gene in this serogroup that might be associated with HUS. Pangenomes of 65 O157:H7 strains consisted of 6,608 genes. Scoary identified a number of accessory genes among O157:H7 strains that were significantly overrepresented in strains from HUS patients (Benjamini–Hochberg adjusted *p* < 0.05) (Supplementary Table S4); however, most of these genes were related to hypothetical proteins whose function remain to be characterized. MCA of pangenomes showed that O157:H7 strains from HUS patients, mostly belonging to clade 8, clustered closely, while strains from non-HUS patients were discretely distributed (Fig. 1C and 1D).

### Phylogenetic relationship of STEC strains from patients with and without HUS

**Fig. 2:**
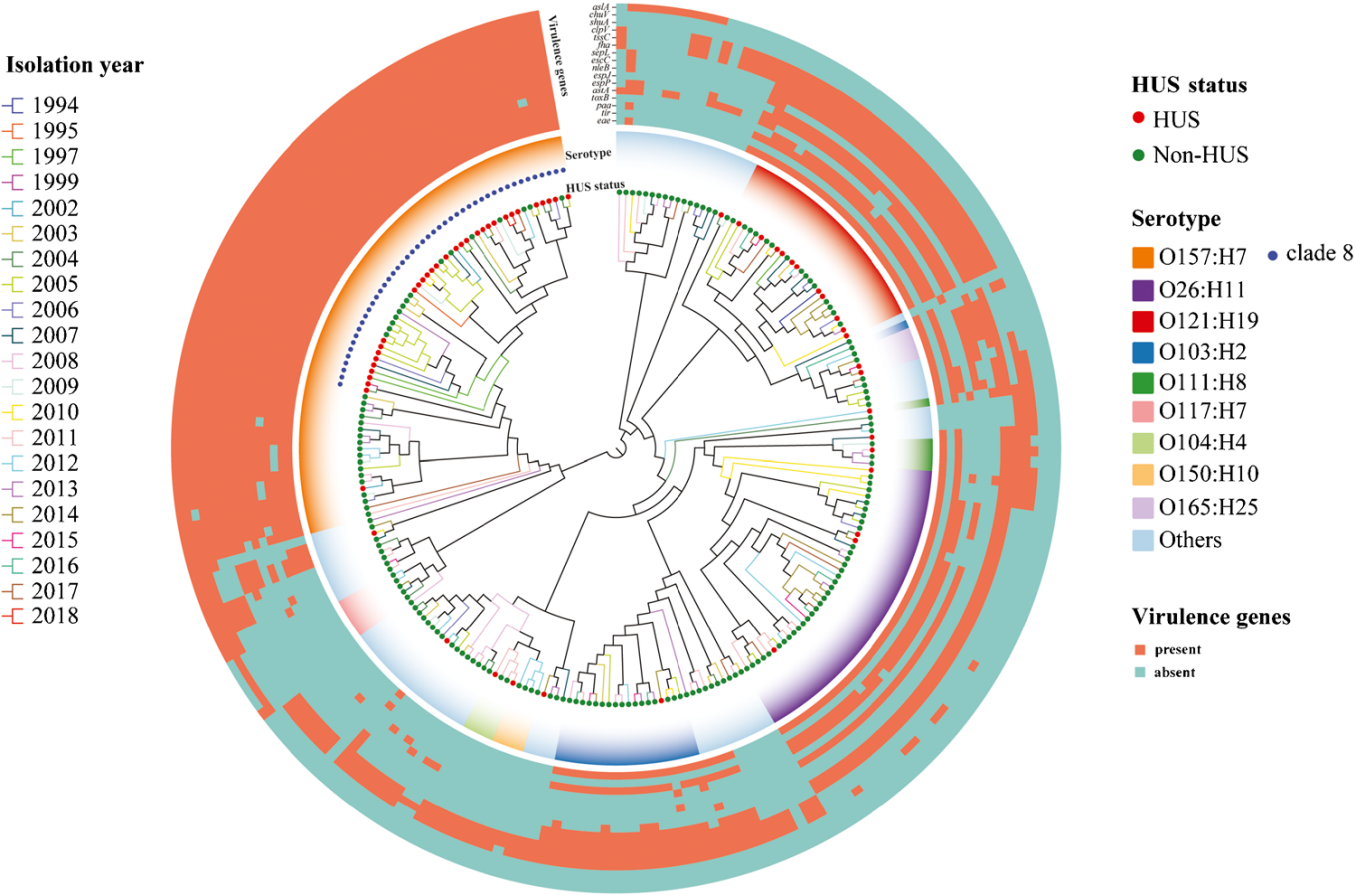
Whole-genome phylogeny of Shiga toxin-producing *Escherichia coli* (STEC) isolates. Circular representation of the Gubbins phylogenetic tree generated from the concatenated sequences of the shared loci found in the wgMLST analysis. Gubbins tree was annotated with relevant metadata using an online tool ChiPlot (https://www.chiplot.online/). The color of branches indicate the isolation year. Branch length is ignored for better visualization. The circle from the inner to outer represents HUS status, serotype (O157:H7 clade 8), and heatmap of representative virulence genes in each functional category that was significantly overrepresented in HUS-STEC strains compared to non-HUS-STEC strains

A whole-genome phylogenetic tree was constructed by alignment of 2,341 shared genes in 238 STEC genomes (Fig. 2). Strains with the same serotype clustered together. In line with MCA of pangenomes, O157:H7 strains were phylogenetically separated from non-O157 strains, and O157 strains of clade 8 were grouped closely. Although no separate cluster was observed for HUS-STEC group, the majority of HUS-STEC strains were distributed on O157 cluster in particular clade 8 and O121 cluster. Strains of same serotype carried similar virulence gene spectrum independent of their HUS status. Genetically closely related strains were isolated from different years.

## Discussion

In this study, we performed a genome-wide association study on a large collection of clinical O157 and non-O157 STEC strains from patients with and without HUS in Sweden between 1994 and 2018. O157:H7 can be classified into nine phylogenetically distinct lineages, as determined by single nucleotide polymorphism genotyping; one lineage (clade 8) was found to be associated with more severe disease such as HUS [27, 28]. The majority of clinical human and bovine isolates belonged to the hypervirulent clade 8 in Argentina [29, 30]. Our study showed that strains of O157:H7 serotype, especially those from the clade 8, were most commonly found in patients with HUS in Sweden. An earlier study showed that clade 8 strains were overrepresented among isolates from cattle farms associated with human cases in Sweden [31]. Shiga toxin gene subtypes *stx2a* and *stx2a* + *stx2c* were found to be significantly associated with development of HUS, while *stx1a* was associated with a reduced risk of HUS, in line with studies from other Nordic countries [13, 14]. Other virulence markers associated with HUS mainly included genes encoding intimin (*eae*), adherence factors, toxins, and type III secretion system proteins. It should be noted that the association observed between bacterial factors and clinical outcomes does not indicate any causal link. Further studies are warranted to examine the functions of these identified genetic makers and their potential roles in HUS pathogenesis. It is notable that there is great geographical variation in genetic characteristics of pathogenic STEC strains that correlates with disease severity. For instance, a recent study from Finland indicated that *eae* was not statistically overrepresented in HUS-STEC strains from pediatric patients, while cytolethal distending toxin (CDT) encoding genes *cdtA*, *cdtB*, and *cdtC* were the most discriminative virulence genes overrepresented in the Finnish pediatric HUS-STEC strains [16]. In the present study, we did not find CDT genes overrepresented in Swedish HUS-STEC strains. Moreover, most HUS-associated O157:H7 strains in Finland were non-clade 8, while all HUS-associated O157:H7 with one exception belonged to clade 8 in this study. Another recent study in Argentina demonstrated no relationship between disease severity and serotypes and genotypes of STEC [32]. These data suggest genetic differences of pathogenic STEC strains in different geographical regions and populations (e.g., age and sex). It may also indicate that non-bacterial factors (e.g., human immunity) and/or bacteria-host interaction play a more important role in STEC-associated disease progression. Future large-scale studies with representative strains and clinical data from various geographical regions and populations are essential to gain further insights.

In the present pangenome-wide association study, we identified a large number of accessory genes differentially presented in HUS-STEC and non-HUS-STEC strains. Besides virulence genes mentioned above, other genes that were significantly overrepresented in HUS-STEC strains mainly encode outer membrane proteins, transcriptional regulators, and phage-related proteins. In addition, numbers of significant genes encode hypothetical proteins (HP) whose functions are poorly understood, further studies are needed to characterize these HP genes and to evaluate their potential role in STEC pathogenesis. Whole genome phylogeny and MCA of pangenomes could not separate HUS-STEC strains from non-HUS-STEC strains, which was in line with earlier studies from Finland and Norway [16, 33]. These results suggest that STEC strains from different phylogenetic lineages may independently acquire genes that determine their pathogenicity. It is noteworthy that O157:H7 strains from HUS patients grouped closely, separated from strains from non-HUS patients. Nevertheless, no significant difference in virulence genes was found between O157 strains from patients with and without HUS, and the majority of significant accessory genes identified at pangenome level were functionally uncharacterized. These data support that other factors, e.g., infection dose of pathogen, variations in host innate, and adaptive immunity, may play an important role in STEC pathogenesis and development of HUS. Further study is warranted to elucidate the host factors in correlation to HUS pathogenesis.

In conclusion, our study revealed that STEC strains of O157:H7 serotype especially clade 8 variants were most commonly found in patients with HUS in Sweden. Genetic factors identified as molecular predictor for development of HUS included *stx* subtype *stx2a*, *stx2a* + *stx2c*, and genes encoding intimin, toxins, secretion system proteins, and transcriptional regulators. Further studies are needed to evaluate the functions of these genes and their role in the development of HUS. Whole genome phylogeny and MCA of pangenomes could not differentiate HUS-STEC strains from non-HUS-STEC strains, suggesting that STEC strains of diverse genetic backgrounds may independently acquire genes that determine their pathogenicity, and that other non-bacterial factors may play a crucial role in the development of HUS, which warrants further investigation.

## Supplementary Information

Below is the link to the electronic supplementary material.

Supplementary file1 (XLSX 202 KB)

## Data Availability

Genome assemblies of STEC isolates were accessible in GenBank with accession numbers presented in Supplementary Table S1.
